# Benefits and risks of staff-owned dogs in small animal clinics: perspectives of employees that bring their dog to work

**DOI:** 10.3389/fvets.2026.1867788

**Published:** 2026-07-06

**Authors:** Elina Herrendorf, Geraldine Holenweger, Helene Rohrbach, Ariane Schweighauser, Claude Messner, Simone Schuller

**Affiliations:** 1 Small Animal Clinic, Department of Clinical Veterinary Medicine, Vetsuisse Faculty, University of Bern, Bern, Switzerland; 2 Department Consumer Behavior, Institute of Marketing and Management, Faculty of Business, Economics and Social Sciences, University of Bern, Bern, Switzerland

**Keywords:** benefits, dogs in the workplace, guidelines, risks, veterinary staff

## Abstract

Many veterinary staff are dog owners, and the opportunity to bring their dog to work may influence their choice of workplace. This qualitative study assessed the benefits and risks for employees who bring their dogs to work in small animal clinics and aimed to develop guidelines to maximize benefits and minimize risks. The study explored the perspectives of employees at four small animal clinics in Switzerland who brought their own dogs to work. Semi-structured interviews were conducted using an interview guide informed by an ecological model encompassing individual, social, community, and societal aspects. In total, 29 interviews were conducted across various job roles and analyzed. Perceived benefits were consistent across the four clinics, whereas the associated risks varied according to organizational structures. Common benefits for the dog-owning staff included reduced stress, increased social interaction, and improved work–life balance. Identified risks included added stress due to care demands of the dog, additional cleaning demands, potential disease transmission, distractions, and organizational challenges related to spatial arrangements for staff-owned dogs. These findings informed the development of practical guidelines for managing staff-owned dogs in the workplace. Key recommendations include clear and consistent communication of rules, the provision of suitable spaces, and structured integration of dogs into the workplace. From a dog-owner perspective, the opportunity to bring dogs to work has the potential to be an important contributor to employee well-being in veterinary settings. The implementation of formalized guidelines may help to create a positive environment for staff, staff-owned dogs, and employers.

## 1 Introduction

Permitting employees to bring their pet dogs to the workplace has been associated with benefits for these employees, including reduced stress and improved work-related quality of life, organizational commitment, and social connectedness (1, 2). In recent years, interest in dog-friendly workplace policies has increased, both as a strategy to support employee well-being and as a potential tool for recruitment and retention (3). This topic is particularly relevant in veterinary medicine, a profession widely recognized for its high demands and long working hours (4).

Veterinary professionals typically have a strong affinity for animals, and dogs often play an integral role in their daily lives. Consequently, workplace policies that allow staff to bring their own dogs may be especially meaningful in this context and may serve as an incentive for attracting and retaining qualified personnel (3, 5). Some job applicants actively seek dog-friendly workplaces, preferring this option over leaving their dogs at home or with caretakers (1). From an employer’s perspective, small animal clinics may also benefit indirectly from the positive effects of dogs on employee well-being documented in non-veterinary workplace settings (1–3).

A recent review examined the benefits and risks of dogs in the workplace for both employees and workplaces (6). The existing literature on dogs in the workplace largely comprises survey- and interview-based studies conducted in office-based environments. Across these studies, employees commonly report lower perceived stress when their dogs are present at work (2, 3). These self-reported benefits are corroborated by physiological data, including reduced salivary cortisol concentrations by performing stressful tasks in the presence of a dog (7). Experimental research further indicates that pet ownership can lower blood pressure response to mental stress (8). Additionally, the option to bring their dogs to the office reduces employees’ stress by enabling them to stay close to their pets throughout the day, which in turn reinforces the owner-dog bond (5). In addition to stress reduction, dogs may also act as social facilitators in the workplace, promoting social interactions, strengthening collegial relationships, and contributing to a more positive workplace atmosphere (3).

Despite these potential benefits for employees, the presence of dogs in the workplace also introduces several challenges for the workplace. Dogs may act as a source of distraction for some employees (9), and although dog owners often report no decline in productivity, the competing demands of professional responsibilities and dog supervision may become burdensome during periods of high workload or in demanding situations (2, 3). Additional concerns include fear of dogs or allergic reactions among staff members (1–3, 9), increased hygiene requirements (2), and safety hazards associated with dog-related objects such as leads, toys, or gates, which may pose risks of trips or falls (3, 10).

Small animal veterinary clinics represent a unique workplace context in which the implications of staff-owned dogs may differ substantially from those observed in office environments. They are workplaces with hospitalized animals, strict indoor and outdoor hygiene requirements and infection prevention and control including vaccination and deworming requirements to prevent spread of infection. In addition, the emotionally challenging nature of veterinary healthcare (4) may amplify the positive effects of bringing one’s companion animal to work on reducing stress, but at the same time may also potentially increase stress through care demands for the dog. Although dogs cannot accompany their owners during demanding work tasks, it may be beneficial for employees to take a break with their dog after a stressful task or to have their dog present during office work.

The present study aimed to investigate the experiences of dog-owning employees in small animal clinics who bring their dogs to work, focusing on the perceived benefits and risks for these employees and the clinics, and to compare these experiences across different organizational models. Based on these insights, the study sought to develop evidence-based guidelines for the integration of staff-owned dogs into small animal clinics, with the objective of maximizing potential benefits while addressing and mitigating associated risks.

## 2 Materials and methods

### 2.1 Ethics statement

The study protocol was reviewed in advance by the Ethics Committee of the Faculty of Business, Economics and Social Sciences at the University of Bern (Reference 362022). They approved the study protocol following ethical standards in accordance with the “Declaration of Helsinki” by the World Medical Association and according to the “Ethical Principles of Psychologists and Code of Conduct” by the American Psychological Association (APA).

### 2.2 Study design

Qualitative data were collected through semi-structured interviews conducted with employees who bring their dog to work at four small animal clinics in Switzerland between May 2023 and February 2024. Two clinics were academic teaching hospitals, and two were large private clinics. The number of guided interviews was determined according to the principle of theoretical saturation, whereby data collection at a given clinic was considered complete once interview responses became repetitive and no new themes emerged (11, 12).

### 2.3 Participants

The clinical management of each clinic was contacted by the study team and invited to participate. In each clinic, all employees who regularly brought their own dog to work were invited to take part, and those who agreed were interviewed. This approach was chosen to interview employees who benefit from being able to bring their dogs to work and who can also provide insights into the potential or perceived risks associated with this practice. To gain a second perspective, members of the clinic management were also interviewed in each clinic, as they are responsible for deciding whether staff-owned dogs are permitted in the workplace and for establishing and implementing relevant policies. Participants represented a range of professional roles and were classified as young clinicians, senior clinicians, veterinary technicians, or administrative staff. All interviewed participants signed a consent form from the Department of Consumer Behavior at the University of Bern before participating.

### 2.4 Interview guidelines and interview process

**Figure 1:**
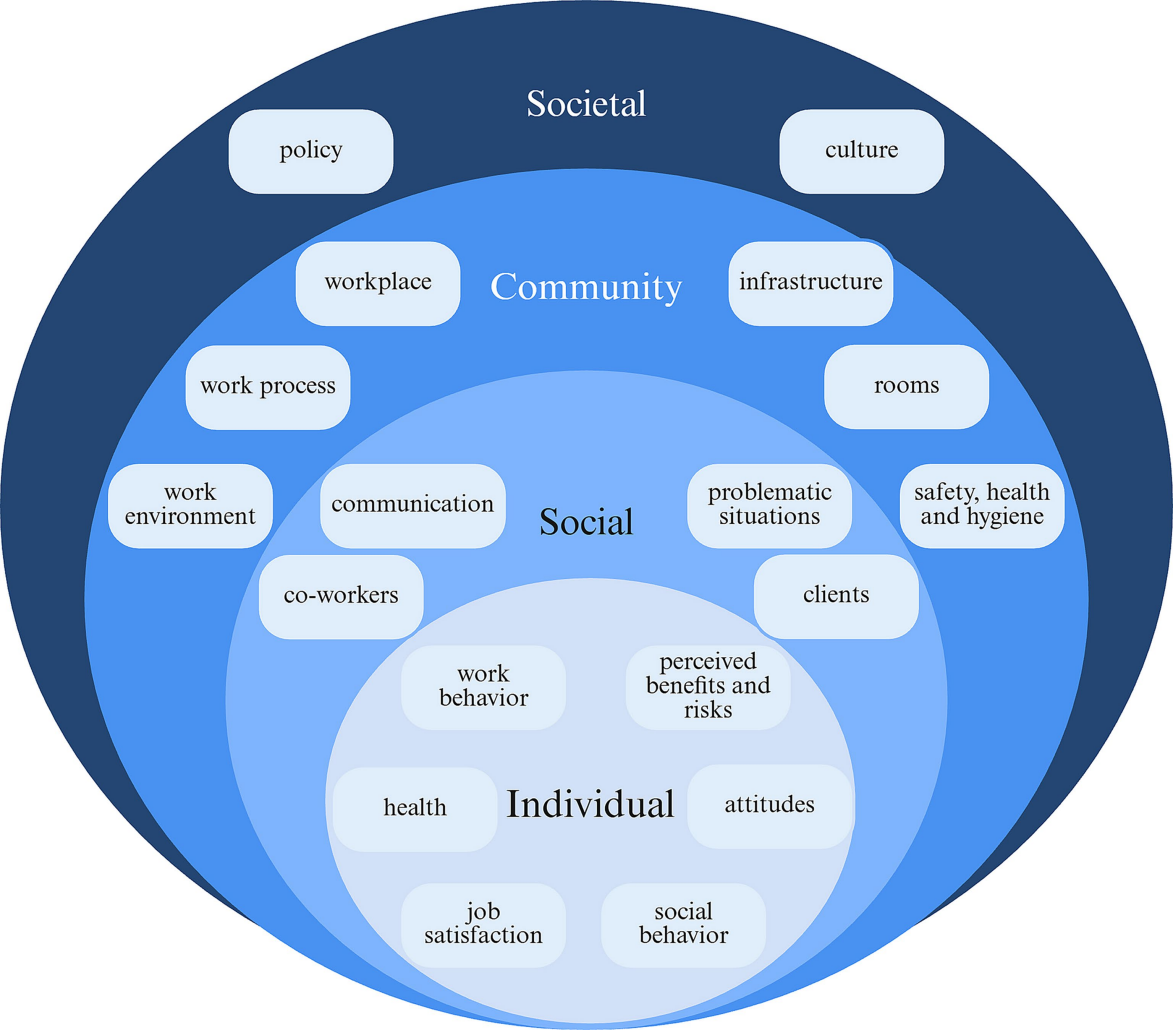
Ecological model adapted from Dahlberg and Krug (13). Created in BioRender. Herrendorf, E. D. (2026) https://BioRender.com/6jxw2fe.

Interview guidelines were developed using the ecological model described by Dahlberg and Krug in 2002 (13). This model draws on ecological systems theory, which assumes that human behaviors are influenced by factors at the individual, relationship, community, and societal levels of an individual’s environment. Possible influencing factors at different levels of the model were identified based on the current literature on the presence of dogs in the workplace (Figure 1).

Open-ended interview questions were formulated based on these factors and grouped into the following key areas.

- Individual level: Impact of bringing a dog to work on the owner, including work behavior, health, job satisfaction, and social interactions, as well as the dog’s well-being, the owner–dog relationship, and workplace integration.
- Social level: Interactions between the dog owner and colleagues and clients, with a focus on problematic situations and social dynamics.
- Community level: Workspace, environment, and infrastructure.
- Societal level: Safety, hygiene, rules, and workplace culture.

Interviews were conducted in person, either at the participant’s workplace with their dog present or in a separate room, with or without the dog. Each session lasted approximately 40 min, was conducted in German, and was audio-recorded. The interview began with an informal introduction to help the interviewer get to know the participant and their dog, following which the guided questions were asked. At the end of the interview, participants were invited to share additional thoughts not addressed in previous questions. The complete interview guidelines are provided in Supplementary file 1. The interviews were transcribed pseudonymously using MAXQDA software (version 22.6.0).

### 2.5 Review of organizational models and clinic policies

Information on the organizational models at the clinics was collected through interviews with clinic management and responsible staff members, as well as through a review of existing policy documents. The data collected included coordination and approval processes, spatial arrangements and allocation procedures, and whether dog owners were required to contribute financially in order to bring their dog to work. Based on this information, the specific benefits and risks associated with each organizational model were identified.

### 2.6 Data analysis

Interview transcripts and clinic policies were thematically analyzed using a codebook-based approach following Braun et al. (11). A preliminary codebook was deductively developed from the interview guide, using ecological model–informed codes (e.g., individual impacts on dog owners’ professional lives). All interviews were initially coded using this codebook, with new codes added inductively, and existing codes refined into subcategories as analysis progressed. The final codebook is available in Supplementary file 2. Coded excerpts generated in MAXQDA were then organized by code. For each clinic, similar responses were grouped to reflect consensus, while contradictory statements were noted. All statements were categorized as either perceived benefits or risks of employees bringing their own dogs to work, for both the employees and the clinic.

### 2.7 Guideline development

**Figure 2:**
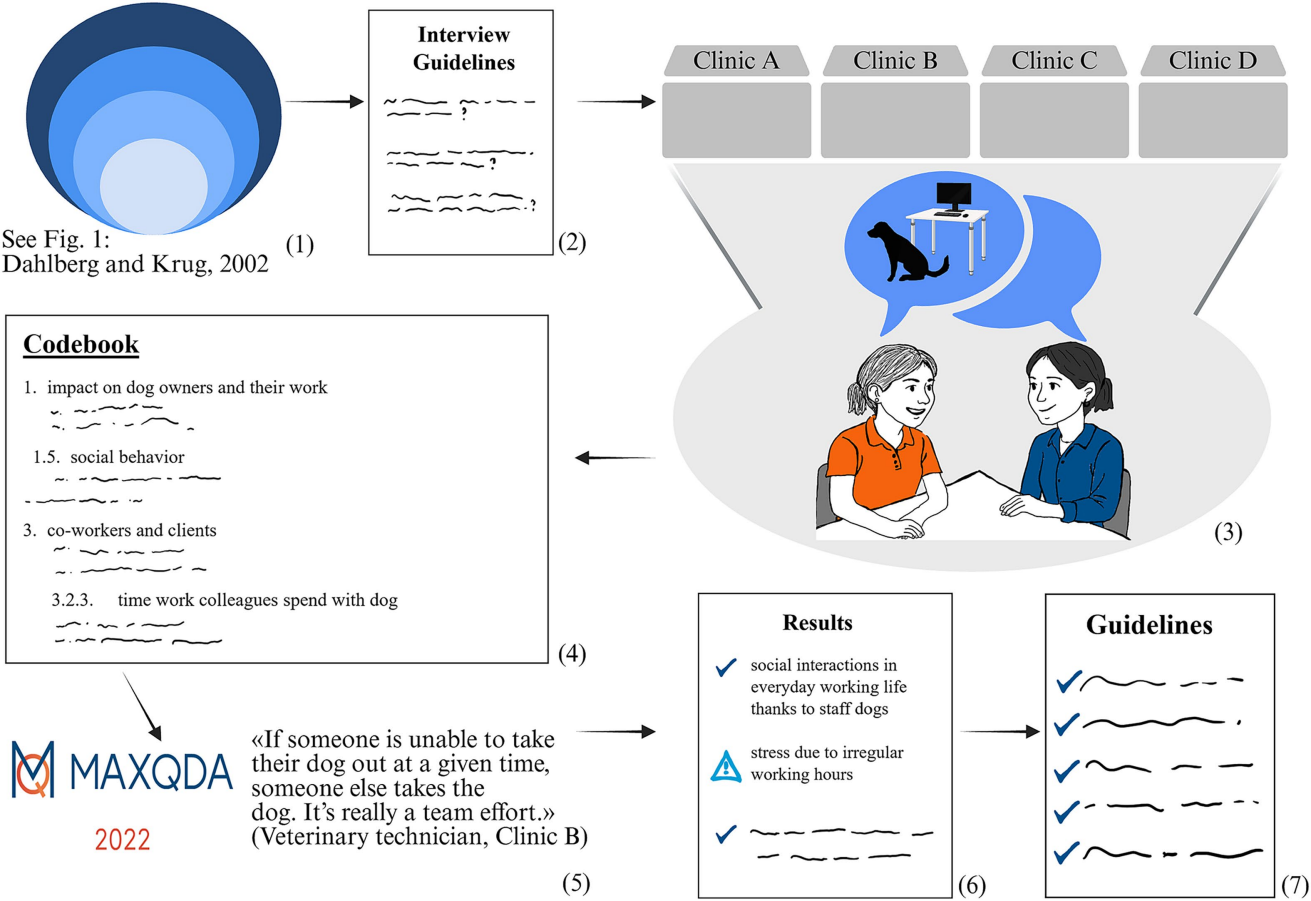
Process for data acquisition, analysis, and guideline development. Created in BioRender. Herrendorf, E. D. (2026) https://BioRender.com/9lm8206.

Based on the analysis of interview data and clinic policy documents, effective practices and risk-mitigation strategies were identified and used to formulate generally applicable guidelines. An overview of the processes of data acquisition, analysis, and guideline development is presented in Figure 2.

## 3 Results

### 3.1 Clinics and participants

A total of 29 participants were interviewed (Clinic A: *n* = 5; Clinic B: *n* = 10; Clinic C: *n* = 8; Clinic D: *n* = 6). Of these, 28 (97%) were female; 16 interviews (55%) were conducted in the presence of the dog. The dog breeds owned by the interviewees were as follows: Nine dogs were mixed breeds, all of which were adopted from dog shelters. Most common breeds were sheepdogs (Border-Collie-Mix, Mini Australian Shepherd, German Shepherd, Dutch Shepherd, *n* = 7), retrievers (Flat Coated Retriever, Labrador Retriever, *n* = 3), hunting dogs (Magyar Vizsla, small Muensterlander, *n* = 2), others were Rhodesian Ridgeback, Newfoundland Dog, Mastiff, Irish Terrier, Lagotto, Dachshund, Chihuahua and Bolonka (each *n* = 1). The mean age of the dogs was 5.3 years (ranged from 9 months to 12 years).

### 3.2 Organizational models

Each of the four clinics had its own organizational models for employees bringing their dogs to work, ranging from very informal to highly structured and institutionalized approaches. Each clinic implemented a distinct model for housing staff-owned dogs, largely determined by the availability of indoor and outdoor space.

At *Clinic A*, staff-owned dogs were managed by a member of the clinic administration who registered all staff-owned dogs being present in the clinic and kept a waiting list if all available places were occupied. Recently, high demand prompted the refurbishment of a neighboring building to accommodate staff-owned dogs. This dedicated dog hall was equipped with containers containing individual crates, allowing dogs to see and hear one another while remaining physically separated. Some owners enriched the environment with pheromone diffusers to enhance their dogs’ comfort. Few staff-owned dogs were also accommodated in offices, if their owners were permanently present in the office. Allocation of the dogs was decided by the clinic management with input of the dog owner. Every dog-owning staff was charged a fixed amount for their dogs’ accommodation.

At *Clinic B*, a dog committee composed of seven members from various staff groups organized the admission of staff-owned dogs to a central outdoor kennel serving clinics and institutes on a larger campus. Upon written application, dogs were allocated by this committee with input of the owner. All staff-owned dogs were registered by the committee, which also maintained a waiting list and supervised that their written regulations about staff-owned dogs at work were followed. All dog owners made a monthly financial contribution which was adapted to the owner’s income. Approximately half of the staff-owned dogs were housed in offices, the other halve was housed in outdoor kennels in small groups of two to four dogs.

At *Clinic C*, anyone could bring their dog to work, provided they found a suitable location in accordance with their superiors. A larger proportion of staff-owned dogs were housed in offices, if co-workers in these offices agreed. A fenced outdoor area was available to well-adjusted staff-owned dogs. Some dogs were also accommodated in unused patient boxes. There were only few rules regarding staff-owned dogs at work, mostly for the outdoor area as it was located near a residential neighborhood, requiring careful management to prevent excessive barking and avoid complaints from neighbors.

At *Clinic D*, dog-owning staff could bring their dogs to the workplace after the management and other dog-owning co-workers agreed, because most dogs were housed collectively in a designated dog room adjacent to the hospitalization area. The intention was to integrate all dogs into the dog group. The door to the dog room had been replaced with a gate. Due to limited space, less socially compatible dogs were also accommodated in offices, in empty patient boxes or consultation rooms.

At *Clinics C* and *D*, both staff and clinic management welcomed an independent organization with few rules; however, a senior clinician from Clinic D was somewhat critical, “Sometimes we think for ourselves, but not for the group.”

Table 1 gives an overview over the different organizational elements of these four clinics.

**Table 1:** Organizational elements in the four clinics.

Clinic	A	B	C	D
Coordination and approval process	Clinic administration	Dog committee decided after the written application of the dog owner	Informal, superior had to agree	Informal, after consultation with management and other dog-owning employees
Space allocation	By clinic management, with input of owner	By committee, with input of owner	By owner, with the consent of office co-workers	By owner, with the consent of co-workers
Limitation	Number of staff-owned dogs limited, waiting list	Number of staff-owned dogs limited, waiting list	No limitation	No limitation
Formal register	All staff-owned dogs registered	All staff-owned dogs registered	No register	No register
Written regulations	Yes	Yes	No	No
Financial contribution	Yes, the same amount for every owner	Yes, depending on owner’s income	No	No
Locations	Extra building with individual dog crates, few office dogs	Offices, outdoor kennels	Offices, fenced outdoor area, unused patient boxes	Offices, dog room in clinic, occasionally empty consultation rooms, and patient boxes

### 3.3 General benefits and risks for dog-owning employees bringing dogs to work and for the clinics

Some benefits and risks for dog-owning employees and the clinics associated with employees bringing their own dogs to work were observed across all four clinics, independent of their organization models. Table 2 presents these general benefits and risks.

**Table 2:** Overview of general benefits and risks.

General benefits	General risks
May reduce stress for dog-owning staff because they can attend to their dog even during long workdays More attractive workplace for dog-owning employees More affordable than day-care Walking dogs helps staff regenerate during breaks Staff-owned dogs can assist in staff training or serve as blood donors Dogs promote social interaction among employees	May increase stress for dog-owning staff due to irregular break times and long working hours Fights between dogs and damage to property Distraction from barking or restless dogs in offices Available kennels not suitable for all dog breeds and characters Increased risk of infection for staff-owned dogs Additional workload for cleaning staff Staff’s fear of dogs

*Impact on dog owners stress perception:* Across all four clinics, employees placed a high value on being able to bring their dogs to work. Many participants reported a lack of affordable or suitable alternatives, such as dog day-care, and preferred not to leave their dogs alone at home due to long commuting times and unpredictable working hours. Employees who bring their dogs to work also reported being more likely to take a lunch break on days when their dogs were present, compared with days when they were not. A veterinary technician stated, “I think I am more relaxed because I have a break during the day. I go into the forest and focus on something else. I am not in this building, or around it, all day.” However, participants reported experiencing stress if they were unable to take their dog out at lunchtime. A veterinary technician explained, “If I only have a 20-min break and have to rush to take the dog outside and then come back, I cannot really sit down anymore; depending on the situation, that can be exhausting.” Many employees preferred not to bring their dogs to work during night or weekend shifts, as balancing their clinical duties and dog care was perceived as difficult during these periods.

*Integration of dogs in the workplace:* Participants across all clinics were very committed to integrating their dogs into their workplace. Staff-owned dogs were, for example, used to practice medical examinations with trainees or served as blood donors. Many interviewees described staff-owned dogs as part of the clinic team. However, conflicts occasionally arose, particularly when dogs damaged clinic property or experienced stress in shared housing arrangements, as a veterinary technician from Clinic A experienced:

[My dog] was in a box with dogs belonging to a good colleague of mine, and they didn’t get along inside that box. Her dogs were extremely stressed, and mine didn’t like that they were stressed. As a result, he would destroy his bed. That’s why I changed the container, and now the problem is solved.

*Increased social interactions:* At all clinics, participants reported that the presence of dogs increased everyday social interactions. Dog owners frequently went on walks together during lunch breaks or after work, and dogs facilitated informal conversations with both dog-owning and non-dog-owning co-workers.

*Distraction from barking or restless dogs in offices:* Some participants from the different clinics mentioned that some colleagues were not particularly fond of dogs in the workplace, though they did not know the exact reasons. They speculated that it might be due to the noise from barking or the commotion in the office.

*Available kennels not suitable for all dog breeds and characters:* Participants emphasized the importance of dogs being familiar with one another prior to grouping and often arranged joint walks before integrating a new dog. Not all dogs or group combinations were considered suitable for this arrangement, necessitating careful placement. Outdoor environmental conditions could be difficult for small or short-coated breeds during winter and for thick-coated dogs during summer.

*Increased risk of infection:* Participants at all clinics recognized the elevated risk of infection associated with bringing staff-owned dogs to work. Across all clinics, staff-owned dogs and patient dogs shared outdoor areas for urination. Employees attempted to minimize their dogs’ exposure time in these areas to reduce infection risk. In addition, open dog food left in dog areas attracted rodents at several clinics, posing an additional hygiene concern.

*Additional workload for cleaning staff:* At all locations, dog owners assumed primary responsibility for maintaining cleanliness in staff-owned dog areas. Clinic D implemented a formal cleaning schedule for the dog room, Clinic C organized regular group clean-up events for the outdoor area, and at Clinics A and B, owners cleaned their dogs’ boxes or kennels individually. Employees who kept dogs in offices were responsible for maintaining those spaces. Despite these efforts, participants acknowledged that the presence of dogs increased the overall workload for cleaning personnel.

*Fear of dogs:* Although most veterinary clinic employees were accustomed to working with animals, some employees might be afraid of dogs, as a young clinician said, “Cleaning staff are not necessarily trained to handle contact with unfamiliar dogs. And some are simply afraid of dogs, which is understandable.”.

### 3.4 Clinic-specific benefits and risks for dog-owning employees bringing dogs to work and for the clinics

Benefits and risks for both dog-owning employees and the clinics that were directly linked to the clinics’ organizational models are summarized in Table 3.

**Table 3:** Overview of clinic-specific benefits and risks.

Clinic	Specific benefits	Specific risks
A	Large number of individual crates	Dog hall unsuitable for some dogs Risk of conflicts among staff from inconsistent rule application
B	Open office doors allow communication among staff to flow Dog community	Path to outdoor kennels via patient area Hospitalized dogs can hear staff-owned dogs and vice versa Risk of conflicts among staff from inconsistent rule application Indirect communication in case of conflicts via the dog committee instead of directly with affected dog owners
C	Suitable outdoor area	Closed office doors, creating segregation or isolation of staff
D	Personal responsibility and few rules Direct communication in case of conflicts Dogs well-occupied in the group	Door gate not escape-proof Dog room close to patient hospitalization Limited space available

#### 3.4.1 Clinic A

*Large number of individual crates:* Recently, high demand to bring staff-owned dogs to work lead Clinic A to use a neighboring building to accommodate them. Before, only few dogs were accommodated in used or unused offices. The new concept with the dog hall gave necessary space for a lot of staff-owned dogs.

*Dog hall unsuitable for some dogs:* However, not all employees used this facility. Some found the crates unsuitable, especially for older dogs unaccustomed to being alone in a crate for prolonged periods.*Inconsistent application of rules as a source of dissatisfaction:* Some veterinarians struggled with the change from few office places to the big dog hall, as they lost the privilege of keeping their dogs in their office. Given the size of the clinic, the management could not offer personalized solutions. Some employees were still allowed to have their dogs in the office, but only if they could permanently supervise their dogs in the office. A veterinarian constated: “There are people who are officially permanently in their office and are allowed to have their dog there. However, they are usually not there… I find that difficult sometimes.”

#### 3.4.2 Clinic B

*Open office doors allow communication amongst staff to flow:* At Clinic B, many dog-owning employees used self-provided door gates to confine their dogs within offices while maintaining accessibility to colleagues. However, these gates constituted a safety hazard, posing a tripping risk and having already been associated with workplace accidents.

*Dog community:* Over time, a sense of community developed among dog owners, as one veterinary technician mentioned, “You help each other out. If someone is unable to take their dog out at a given time, someone else takes the dog. It’s really a team effort.”

*Path to outdoor kennels via patient area:* Staff preferred to access outdoor kennels via the clinic building rather than crossing an outdoor area perceived to have higher contamination with potential pathogens. A veterinary technician remarked, “The inner corridor is cleaned and disinfected daily. But patients are walked outside […] so many times a day, and there, it is not often cleaned.”

*Hospitalized dogs can hear staff-owned dogs and vice versa:* Hospitalized patients were exposed to the sound of barking staff-owned dogs in the outdoor kennels, which could further increase stress in already stressed patient animals. Conversely, staff-owned dogs also heard hospitalized patients barking, which in turn might have disturbed them. One veterinary technician explained: “I can imagine that it is stressful for patients when a dog is barking. On the other hand, some patients bark for hours as well. That is not pleasant for the other patients either.”

*Inconsistent application of rules as a source of dissatisfaction:* Clinic B had written policies governing staff-owned dogs in the workplace. However, several dog-owning employees reported that the rules were not always applied consistently. This inconsistency contributed to perceptions of inequity and dissatisfaction, as a veterinary technician explained, “Many veterinarians have their dogs in their private offices, while veterinary technicians don’t have access to an office. They must keep their dogs in the outdoor kennels.”

*Communication culture in conflict situations:* By contrast, several employees at Clinic B reported that concerns related to staff-owned dogs were rarely addressed through direct communication. Instead, non-dog-owning employees raised issues, such as disturbances caused by barking, with the clinic’s dog committee rather than with the dog owner directly. The committee then responded by sending general email reminders to all dog owners. Participants noted that these non-specific messages sometimes caused unnecessary concern among those not directly involved. Dog owners expressed a preference for a more direct and open communication culture.

#### 3.4.3 Clinic C

*Suitable outdoor area:* The fenced outdoor area in Clinic C was divided into three groups for a limited number of dogs. Dog areas were equipped with doghouses, straw and a grassy area. Participants mentioned their dogs being well-exercised and socialized within their group. The fenced outdoor area was highly valued by employees, as a senior clinician pointed out:

At worst, you can leave him outside for eight hours. Then you know: he can pee, he can do his business, he might be a little bored, which isn’t ideal. But at least he isn’t trapped in an office and in distress.

*Closed office doors:* Most dog-owning employees kept their office doors closed because many dogs became restless and barked when people walked by. A member of the administrative staff mentioned, “I must always keep the door closed so that my dog doesn’t bark […]. That makes me feel a bit isolated.”.

#### 3.4.4 Clinic D

*Personal responsibility and few rules:* Dog-owning staff at Clinic D valued the freedom to manage their dogs by their own as they knew their needs best. They did not want more rules. However, open communication was important to them so that conflicts could be addressed.

*Direct communication in case of conflicts:* Efforts were made to find individualized solutions for each dog-owning employee. When conflicts arose, they were addressed through direct discussions, weekly meetings, or a monthly newsletter.

*Dogs well-occupied in the group:* In the dog room, dogs interacted socially or rested together. However, older, unwell, or socially incompatible dogs required individual accommodation in offices or other spaces, which was challenging given the limited space.

*Door gate not escape-proof:* The door to the dog room was removed and replaced with a gate. However, some dogs were able to escape, roaming the clinic, leading to repeated complaints from staff working in neighboring areas.

*Dog room close to patient hospitalization:* The proximity of the dog room to the hospitalization area meant that dogs crossed patient areas when entering and exiting the room. This arrangement had already resulted in the spread of kennel cough, a highly contagious infection, among staff-owned dogs. Furthermore, it was perceived that barking staff-owned dogs may negatively impact on hospitalized patients, which might get frightened or excited.

*Limited space available:* If possible, new staff-owned dogs should be kept in the dog room. The clinic tried to allow all employees to bring their own dog to work despite space limitations. A veterinarian explained: “This dog room is limited in size. We have a lot of staff who own a dog […]. That’s where we’re starting to run into some limitations, which is why the dogs are now spread out a bit everywhere.”

### 3.5 Guidelines on bringing staff-owned dogs to work

Based on the findings of this study, guidelines were developed to enhance benefits and minimize risks for both employees who bring their dogs to work and the clinics. Ideally, these guidelines should be considered and appropriate measures implemented prior to allowing staff-owned dogs at a small animal clinic.

#### 3.5.1 Responsibilities of the dog owner

- Dog owners are responsible for the familiarisation, socialisation, and overall welfare of their dog and must ensure appropriate behavior in the workplace. This includes actively preventing conflicts between their dogs, people, and/or other animals.
- Dog owners must also ensure that their work schedule, including lunch breaks, allows sufficient time to meet their dogs’ needs, such as regular walks, supported by clear time structures defined by the employer.

#### 3.5.2 Behavioural and practical requirements for staff-owned dogs

- Staff-owned dogs must not display aggressive behavior toward people or other animals and should remain calm while at the workplace.
- Dogs must be accustomed to being left alone in their designated resting area without their caregiver. Situations likely to trigger excessive barking should be anticipated and managed through appropriate training or supervision.
- Any damage to furniture or infrastructure must be prevented.

#### 3.5.3 Responsibility and organization of the clinic

- All rules regarding staff-owned dogs must be clearly defined and should be communicated in writing. The rules must be applied consistently and clearly understood by all employees.
- If the clinic permits staff-owned dogs in the workplace, it shall provide designated dog areas within its capacity. The maximum number of staff-owned dogs allowed in the clinic at any one time must be defined in advance.
- The clinic shall establish a dog management structure to coordinate and oversee the presence of staff-owned dogs in the workplace. Responsibility may lie with clinic management, designated supervisors, or an appointed dog committee.
- To ensure effective infection control and workplace safety, the clinic must maintain an updated record of which staff-owned dogs are present, where they are located, and their scheduled times in the clinic.
- A defined settling-in period may be implemented to allow staff-owned dogs to acclimatise to the workplace and to ensure that behavioural and practical requirements are met before full integration.
- The clinic may require a financial contribution to cover costs associated with staff-owned dogs in the workplace, taking salary categories into account. The purpose and use of these funds must be communicated transparently to employees.

#### 3.5.4 Dog areas

- A maximum number of dogs per office should be determined based on local animal welfare regulations and the specific needs of the people working in that office.
- Each dog must have a clearly defined area within the office.
- In accordance with local animal welfare regulations, dogs may not be kept on a lead or confined in crates in offices for extended periods.
- Employees must be able to access offices at any time without provoking excessive reactions from the dog. Any installed door gates should be escape-proof, easy to use, and high enough to prevent staff from stepping over them, avoiding tripping hazards.
- Dogs may be kept in small groups in a separate room within the workplace, provided they are well socialized and regularly supervised by their owners.
- If dogs are kept outside, they must be adequately protected from the weather, in line with local animal welfare regulations.

#### 3.5.5 Safety and hygiene

- The dog owner must be able to provide proof of liability insurance for their dog. For safety reasons, staff-owned dogs must be kept on a lead throughout the clinic area.
- To ensure hygiene and not further increase the stress of patient animals, a clear spatial separation must be maintained between patient animals and staff-owned dogs. Staff-owned dogs are not allowed to be kept in patient kennels. If a staff-owned dog is infected, it should not be brought to the workplace. If it requires medical treatment, it must be cared for as a patient in the patient area.
- In the outdoor area, designated patient-dog toilet areas should be established, which are not used by staff-owned dogs. Conversely, separate areas should be defined for walking staff-owned dogs.
- Staff-owned dogs must receive prophylactic treatment against endo- and ectoparasites and be vaccinated in accordance with local vaccination recommendations.
- To prevent rodent damage, dog food must be stored in a sealed container.
- Additional cleaning work, including its organization and responsibilities, must be clearly defined and implemented.

#### 3.5.6 Communication

- Topics related to staff-owned dogs should be regularly discussed among dog-owning and non-dog-owning staff to foster direct communication. Conflicts regarding staff-owned dogs in the workplace should be addressed directly between the parties involved. If no solution can be reached, the dog management structure may be consulted. Mutual consideration within the team and transparent, direct communication can help identify and resolve conflicts at an early stage.
- The formation of a dog community can be encouraged. Staff-owned dogs can promote social contact between employees, enabling dog-owning staff to interact more easily and strengthening workplace relationships.

## 4 Discussion

Most existing research on staff-owned dogs in the workplace has focused on office-based settings. The present study extends this literature by examining the perceived benefits and risks of allowing staff-owned dogs in small animal clinics, drawing on interviews with employees who bring their dog to work at four small animal clinics. We focused on the benefits and risks for the employees who bring their dog to work and the organization a whole. While the well-being of the staff-owned dogs was also addressed in the interviews, it was not the focus of this study.

The primary objective was to inform the development of general guidelines that maximise potential benefits while mitigating associated risks.

All four invited clinics agreed to participate, and both management and staff expressed strong interest in the study. This engagement underscores the relevance of this topic within the small animal veterinary profession.

Several consistent positive patterns emerged across clinics. The presence of staff-owned dogs was associated with increased social interactions among both dog-owning and non-dog-owning employees. Dog-owning staff frequently used their lunch breaks to walk their dogs, often together with other dog owners, fostering a sense of community. These breaks also facilitated physical activity and time outdoors, allowing employees to return to work feeling more refreshed. Such benefits are consistent with findings from office-based studies on dogs in the workplace (1, 3).

Dog-owning employees interviewed in this study described their dogs as integral to their daily lives. Many reported having no suitable alternative care arrangements during long and unpredictable working days and indicated that they actively sought employment in clinics that permitted dogs. From an employer’s perspective, dog-friendly workplace policies may therefore enhance the attractiveness of small animal clinics and support the recruitment and retention of qualified staff.

Across all clinics, the successful integration of staff-owned dogs enabled their inclusion as part of the workplace team. Clinics benefited from staff-owned dogs in several ways: they supported the onboarding and practical training of new clinical staff and, in some cases, served as blood donors for hospitalized patients. In academic settings, staff-owned dogs were also commonly used for hands-on student training and as healthy controls in clinical trials (14). As a result, even employees who did not bring dogs to work could indirectly benefit from the presence of staff-owned dogs.

Despite these advantages, bringing dogs to work also introduced notable risks. Integration required time and careful consideration of individual dogs’ temperaments and backgrounds. Housing arrangements varied widely, including offices, outdoor kennels, and group housing in designated dog rooms. Dogs housed in offices could disturb colleagues through barking or movement, consistent with reports from office-based studies (9). Dogs housed close to hospitalized areas could disturb already stressed patient animals through barking. Limited space within clinics often necessitated creative solutions, such as external kennels or adjacent buildings. The lack of suitable facilities led to less-than-ideal situations, for example the use of patient facilities for staff-owned dogs. Outdoor housing, in turn, posed challenges for dogs poorly adapted to weather conditions. These findings suggest that purpose-built veterinary clinics should explicitly plan and allocate appropriate facilities for staff-owned dogs. For existing facilities, efforts should be made to guarantee welfare standards and to ensure that the housing arrangements do not have a negative impact on the dogs; for example, that the dogs in the housing get along well with one another.

Employees consistently reported that leaving their dogs at home unattended was stressful. However, stress could also arise from managing dogs at work, particularly during periods of high workload. It is relatively common for veterinary staff to forgo their breaks due to a high workload or a critical patient. Staff reported that in situations in which they could not take regular breaks, the need to walk their dog added to their existing stress. Wagner and Pina e Cunha showed that flexible working hours and greater autonomy in scheduling can help dog-owning employees to take care of their dog during work, thus mitigating this risk (3). This strategy can only be partially applied in clinic settings where tight rosters generally do not allow for flexible working hours. However, the veterinary profession is characterised by exceedingly high levels of burnout and suicide (4). Beyond regulatory compliance with mandatory break periods, there is an urgent need for cultural change within veterinary clinics to foster healthy work practices and long-term sustainability. Under appropriate conditions, allowing staff-owned dogs at work may contribute positively to this effort.

Additional risks in small animal clinics include disease transmission, particularly where staff-owned dogs share access routes with hospitalized patients. Both direct and indirect cross-infection between staff-owned dogs and patients is possible. Veterinary clinics have recently been identified as important sites for the selection and dissemination of multidrug-resistant organisms (15). Furthermore, classic canine pathogens, such as canine parvovirus or agents involved in the canine infectious respiratory disease complex (kennel cough), may be transmitted if staff and patient dogs share environments (16), as it already happened in two of the visited clinics. Mandatory vaccination of staff-owned dogs is therefore essential, and spatial separation between staff-owned dog and patient areas is strongly advised. Where separation of access routes is not possible, enhanced cleaning protocols and monitoring are warranted.

The presence of dogs also increases general cleaning requirements and necessitates careful organizational management to prevent conflicts between dog-owning and non-dog-owning staff. In two clinics, dog-owning employees contributed financially to cleaning and maintenance costs. While office workplaces may also experience increased cleaning demands (2), the risk of disease transmission is considerably lower in the absence of animal patients. Conversely, certain risks common in office settings – such as fear of dogs or allergic reactions – were less prominent in veterinary clinics, where staff are generally accustomed to working with animals (1).

All clinics had developed some form of organizational structure and regulation to accommodate staff-owned dogs, typically requiring additional resources and staff time. To minimize conflict, defined rules that are clearly communicated and consistently enforced are essential. Furthermore, fostering a culture of open and direct communication between staff members and management is important to addressing concerns proactively and resolving conflicts in conjunction with the presence of staff-owned dogs at work constructively.

This study has several limitations. Only the perspectives of clinic management and dog-owning employees who brought their dogs to work were considered. The inclusion of views from non-dog-owning staff and dog owners who chose not to bring their dogs to work may have revealed additional benefits and risks. Furthermore, 28 of the 29 interviewees were female. Participation was voluntary, and the study team did not have access to the overall gender composition of staff at each clinic, nor to the proportion of male versus female employees who owned dogs or brought them to work; we therefore cannot determine whether men were less likely to own dogs, less likely to bring them to work, or less likely to volunteer for an interview. Previous questionnaire-based research on workplace dogs has also reported predominantly female samples (5), which may be partly explained by a higher response rate among females rather than the topic itself. However, in this study, this imbalance likely reflects the female-dominated workforce (veterinarians, veterinary technicians, and administrative staff) in small animal clinics in Switzerland. Since women tend to report stronger attachment to and greater empathy toward companion animals than men (17, 18), the near-exclusive female sample may have shaped which experiences were reported and how strongly they were expressed, particularly regarding the emotional benefits of canine company and the stress of leaving a dog at home. The resulting benefits and risks, and the guidelines derived from them, may therefore not fully reflect the experiences of male dog-owning staff. Finally, only four clinics were included, and other organizational models for staff-owned dogs in small animal clinics may exist. Future research should also examine the well-being of staff-owned dogs themselves in these workplace settings.

## 5 Conclusion

Allowing veterinary staff to bring their dogs into the workplace can meaningfully contribute to employee well-being and foster a positive environment for staff, staff-owned dogs, and employers alike. However, risks such as added stress, unsuitable housing solutions, increased infection risk and additional cleaning work should be taken in consideration. The guidelines developed in this study may assist clinics in optimizing the integration of staff-owned dogs, enhancing benefits, and mitigating risks for both dog-owning employees and clinics. For clinics that do not currently permit dogs at work, these guidelines provide a structured framework for a safer and more sustainable implementation of staff-owned dog policies allowing dogs at the workplace.

## Data Availability

Collected data are not publicly available due to concerns about individual privacy, since conclusions can be drawn from the qualitative interviews about the identities of those who made the statements. Excerpts from the data may be requested from the corresponding author in accordance with specific ethical and data protection regulations.

## Supplementary material

SUPPLEMENTARY FILE 1 Interview guidelines that were developed to interview the participants.

SUPPLEMENTARY FILE 2 Codebook that was created to code the interview transcripts.
