# Supplementary material for: Benefits and risks of staff-owned dogs in small animal clinics: perspectives of employees that bring their dog to work
Source: Front Vet Sci. 2026 Jul 6;13:1867788. doi: 10.3389/fvets.2026.1867788 (PMC13383378; doi:10.3389/fvets.2026.1867788)
Supplement: SUPPLEMENTARY FILE 1 — Interview guidelines that were developed to interview the participants. [file Supplementary_File_1.docx]

# Supplementary File 1 – Interview guidelines

### Context of the interview

- Introduction of the interviewer
- Presentation of the project: Interdisciplinary project together with the small animal clinic, focusing on the benefits and risks of bringing your own dog to work in a small animal clinic.

### Organizational information about the following interview

- The purpose of this project is to discuss what experiences have been made with staff dogs in a workplace that allows staff dogs. More specifically, it looks at the benefits, but also the potential risks of bringing a staff dog in the workplace. Both positive experiences and challenges are of interest. It is not about banning staff dogs in the workplace.
- The data collected from the interviews will be published in the form of an exploratory scientific article.
- I would like to record the conversation so that everything is reported correctly.
- Confidentiality is very important to us, that means your answers are not traceable back to you later.
- You can read more about this in the consent form. Please read it through and sign it.
- If it's okay for you, I will take a photo of your dog during this time?
- The interview will take about 45 minutes, if you do not want to answer a question (on the audio recording), just say so.
- Do you have any questions?

**[start audio record]**

## Getting know the interviewee and the dog (5-10 min)

### Please start by talking about yourself

- How long have you been working here?
  - Possible follow-up questions: Where did you work before? Did you also take your dog with you to work there? If yes, what experiences did you have there with your dog at work? If not, why not?
- What does a typical working day look like for you?
  - Possible follow-up questions: % work at the office desk? Workload in %?

### Now please tell us about your dog

- How old is your dog and how long has he been with you?
  - If not yet known: Name and breed of the dog?
- Why did you decide that you wanted a dog?
- How many days a month does your dog come to work with you? Does this depend on certain circumstances? (Examples: other dog care options, special features of the working day)
- When did you start bringing your dog to work? Why since then?
- In what way has there been an exchange between you and your employer regarding bringing your dog to work?

### As an introduction to the topic

- What is your personal attitude towards dogs at work?
  - Possible follow-up question: Towards staff dogs in your workplace?

## Individual: Impact on dog owner and their work (10 min)

We will first discuss the benefits and risks for yourself and your work.

### Work behavior

- Can you tell me about how your work behavior changes when you bring your dog to work (compared to when you don't)?
- If your dog is present, would you consider your focus at work to be lower or higher as a result? Why?
- If your dog is present, would you consider your productivity to be lower or higher as a result? Why?
- Can you tell me how bringing your dog with you affects your work schedule?
  - Possible follow-up questions: Do you take more regular breaks as a result? Does it mean you start work earlier or later? Does it mean you go home earlier or later?

### Health

- Can you tell me about how you get more (or less) exercise during a typical working day when you bring your dog with you?
- How do you get more (or less) time outdoors when you bring your dog to work?
- Does bringing your dog to work tend to reduce or increase stress? Please try to explain which factors tend to reduce stress and which tend to increase stress.
- Can you tell me how bringing your dog to work increases or decreases your wellbeing or quality of life?

### Job satisfaction

- How does bringing your dog to work affect your work motivation?
- What circumstances lead you to be satisfied or dissatisfied with your current work situation?
- How would you react if you were no longer allowed to bring your dog to work?

### Social behavior

- Can you tell me about how bringing your dog to work helps or hinders your social interactions at work?
  - Possible follow-up questions: Are you more likely to get involved in conversations at work because of your dog? How do these conversations arise?
- How does bringing your dog to work affect your relationship with your work colleagues?

## Individual: Wellbeing of the dog (5 min)

Now it's time to talk about your experiences with your dog at your workplace.

### Benefits and risks: Dog

- What does a typical day at work look like for your dog?
- What is the most frequent way you take in the building to get to your dog's place at work?
- Are there places in the immediate environment of the small animal clinic (on the campus) where you spend longer periods of time with your dog? For example, for urinating or playing?
- How does your dog benefit from being at work with you?
  - Possible follow-up questions: In what way does he enjoy meeting other people? How does he enjoy meeting other dogs (or other animals)?
- How can it be stressful for your dog that you take him to work with you?
  - Possible follow-up questions: In which situations does your dog seem to be stressed at work? On a typical working day, how often and in what situations does your dog get time to play and let off steam?

### Owner-dog relationship

- Can you tell me about how bringing your dog to work affects the relationship between you and your dog?

### Integration of dogs in the workplace

- What works well and what doesn't work so well when integrating your dog in the workplace?
  - Possible follow-up question: What could be improved?

## Social: Work colleagues and clients (5-10 min)

Now it's about what experiences you (and your dog) have had with other people at your workplace.

### Problematic situations

- Have you ever experienced a situation where there was a problem at work because of your dog? If so, can you please explain the situation in more detail?

### Work colleagues

- Can you tell me about how your work colleagues react to your dog?
- Can you describe the attitude(s) you know of your work colleagues towards bringing staff dogs to work?
- Can you describe situations in which colleagues have complained about your dog?
  - Possible follow-up questions: To what extent have there been complaints...
    - about disturbances of the peace (example: due to loud barking)?
    - because your dog has caused damage to property? (Example: chewed on something)
- How can your colleagues benefit from you bringing your dog to work?
  - Possible follow-up questions: How does bringing your dog to work change the working atmosphere? In what way do your work colleagues seem to be less or more stressed by bringing staff dogs in the workplace?
- In what situations do your colleagues spend time with your dog? (Example: taking him for a walk)
  - Possible follow-up question: How do these situations arise?
- Can you tell me how your work colleagues behave differently when you have your dog with you?
  - Possible follow-up question: To what extent are they more friendly/open-minded or less friendly?

### Clients

- How do clients react to you (or other employees) bringing staff dogs to your workplace at the veterinary clinic?
- Have there been situations in which clients have complained about your dog (or the dog of a work colleague)? Please describe these situation(s).

## Community & societal (workplace, environment, culture, rules) (10min)

The last part is about the broader context.

### Workspace

- How do you find your workplace suitable or unsuitable for bringing your dog to work?

### Work environment

- Have there been any incidents where your dog or its items (e.g. toys, water bowl) have got in someone's way? Please explain what exactly happened.
- Did your dog cause any damage to property in the workplace? (Example: bitten objects) Please explain what exactly happened.

### Work processes

- How are your typical work processes suitable for bringing your dog to work?
- How are your typical work processes not suitable for bringing your dog to work?

### Safety, health & hygiene

- How do you assess the risk of disease transmission from patients to your own dog?
- How do you assess safety concerns about dogs in the workplace? (Examples: biting behavior, fights with other dogs)
- Have there been any complaints about the cleanliness or hygiene of dogs in the workplace? Please explain how this came about. (Examples: urine, hair, dirt, unpleasant odor)

### Policies & culture

- Do you know any written rules about bringing your dog to work in your workplace? If yes, what is your opinion on these rules?
- In what sense does your workplace have non-established (“lived”) rules for bringing your own dog to work?
- How do these written or “practiced” rules apply to employees from all occupational groups?
- To what extent is bringing your own dog to work a “lived culture” in your workplace?
  - Possible follow-up questions: To what extent have you noticed changes in this “lived culture”? Where/when/with whom? How do you explain these changes?

## Any further questions specific for the clinic

## Conclusion (5 min)

- We have now discussed a lot about dogs in the workplace. Are there any topics that we haven't covered yet that you think are important when it comes to dogs in the workplace?
- Then I would like to thank you very much for your time and effort. You have made a valuable contribution to our study.
