# Supplementary material for: Benefits and risks of staff-owned dogs in small animal clinics: perspectives of employees that bring their dog to work
Source: Front Vet Sci. 2026 Jul 6;13:1867788. doi: 10.3389/fvets.2026.1867788 (PMC13383378; doi:10.3389/fvets.2026.1867788)
Supplement: SUPPLEMENTARY FILE 2 — Codebook that was created to code the interview transcripts. [file Supplementary_File_2.docx]

**Supplementary File 2 – Codebook**

| **code** | **description** |
| --- | --- |
| 1. impact on dog owners and their work |  |
| 1.1 employment contract | how long have they been working there, in which department, what percentage |
| 1.2 work behavior |  |
| 1.2.1 everyday working life | typical day-to-day work, changes with/without dog |
| 1.2.2 work planning | planning with/without dog, e.g. breaks, morning, midday, evening |
| 1.2.3 focus & productivity | work focus, productivity, changes with/without dog |
| 1.3 impact on health |  |
| 1.3.1 pos: dog is doing well (here) | certainty that the dog is doing well, in general or specifically here at work |
| 1.3.2 pos: exercise, nature, outdoors | more exercise in everyday working life, more time in the fresh air, getting outside |
| 1.3.3 pos: stress reduction, psyche | increased wellbeing and quality of life |
| 1.3.4 pos: work and environment | stress-reducing factors relating to work and work environment |
| 1.3.5 neg: concerns wellbeing of the dog | worries about wellbeing of the dog, worries or stress about characteristics of the dog |
| 1.3.6 neg: concerns location of the dog | worries or concerns about the dog's place of residence |
| 1.3.7 neg: own requirements | not meeting own needs and demands, less exercise in everyday working life, less time in the fresh air |
| 1.3.8 neg: work and environment | stress-inducing factors relating to work and work environment, potential for conflicts |
| 1.4 job satisfaction | personal attitude towards dogs in workplaces, work motivation, satisfaction with job situation, reaction to a ban on taking dogs along |
| 1.5 social behavior | social interactions in the workplace, relationship to work colleagues related on interactions of the interviewee |
| 2. wellbeing of the dog |  |
| 2.1 anamnesis of the dog |  |
| 2.1.1 anamnesis of the dog | age, breed, since when at the owners' place, health condition, therapy with antibiotics, vaccinations and deworming, feeding, other animals at home |
| 2.1.2 days a month at work | days a month at work |
| 2.2 dogs’ location and ways it uses |  |
| 2.2.1 office | place of residence in the office |
| 2.2.2 outdoor kennels | place of residence in the outdoor kennel |
| 2.2.3 in the building | which ways used in the building, stays in the building |
| 2.2.4 used entrances and exits | main entrance, entrance next to patients' places, others |
| 2.2.5 outdoor area | which ways used in the outdoor area, stays in the outdoor area |
| 2.3 benefits dog at work |  |
| 2.3.1 social contacts with dogs and people | positive interactions with other dogs or people |
| 2.3.2 suitable dog location | dog feels comfortable at his place, is relaxed, looks forward to being there |
| 2.3.3 occupation of the dog | dog has variety, learns something, has positive stimuli, is busy |
| 2.3.4 closeness dog to owner | closeness dog to owner, to be distinguished from code 2.5.2 (where not necessarily a benefit for the dog) |
| 2.4 risks dog at work |  |
| 2.4.1 insecurities with dogs and people | insecure with other dogs, with people or when alone, defending territory, barking, sensing the influence of sick animals |
| 2.4.2 unsuitable dog location | stressful situations due to location (office or kennel) |
| 2.4.3 further risks | health problems (somatic, psycho-emotional), (past-related) insecurity, excitement due to presence/absence of owner, commute, less exercise than required |
| 2.5 owner-dog relationship |  |
| 2.5.1 Why did owner want to have a dog? | why did owner want to have a dog: in general, this specific dog that owner has now |
| 2.5.2 closeness and time for the dog, training | how is the relationship, closeness owner to dog, time with dog, training, impact on relationship by bringing dog to work |
| 2.6 integration of dog in the workplace | what works well for the integration (everything about integration that does not fit to another code, i.e. work colleagues or risks dog, focus on dog) |
| 2.7 typical daily working life of dog | at work |
| 3. co-workers and clients |  |
| 3.1 problematic situations | problematic situations in the workplace because of dog (situation is seen as a problem by the interviewee, there was communication about the problem) |
| 3.2 work colleagues |  |
| 3.2.1 reaction work colleagues to dog | reaction of work colleagues to dog, if it is/is not at work, positive and negative reactions, also complaints about dog |
| 3.2.2 attitude of work colleagues regarding dogs in the workplace | attitude of work colleagues regarding dogs in the workplace, positive, negative and neutral opinions |
| 3.2.3 time work colleagues spend with dog | time spent from work colleagues with dog: voluntary, by chance, on request |
| 3.2.4 superiors, clinic management | reaction of superiors / clinic management to dogs, their attitude and how they deal with the topic of dogs in the workplace |
| 3.3 clients | reaction of clients and patient dogs to staff dog, encounters, complaints |
| 4. community and societal |  |
| 4.1 rooms |  |
| 4.1.1 office | in relation to the office: suitable rooms, furnishings, advantages and disadvantages of the rooms, possibilities for improvement |
| 4.1.2 outdoor kennel | in relation to the outdoor kennel: suitable rooms, furnishings, advantages and disadvantages of the rooms, possibilities for improvement |
| 4.1.3 other | in relation to other rooms: suitable rooms, furnishings, advantages and disadvantages of the rooms, possibilities for improvement |
| 4.2 work environment | disturbing objects from the dog, property damage caused by the dog (even if own property is destroyed) |
| 4.3 work process (department) | suitability of your own work processes in the relevant department to take dogs with you |
| 4.4 safety, health and hygiene |  |
| 4.4.1 safety concerns | safety concerns of the own dog towards other dogs (private ones, patients) or humans (i.e. fights) |
| 4.4.2 risk of infection | risk of infection with patient animals, hygiene of other dogs |
| 4.4.3 hygiene, cleanliness of own dog | i.e. «I pay attention that I clean my dog on rainy days.» |
| 4.5 policies and culture |  |
| 4.5.1 rules | known rules, practiced rules |
| 4.5.2 application of rules | following the rules, exceptions, dissatisfaction due to inconsistent application of the rules |
| 4.5.3 culture | changing of the culture of bringing dogs to work over time, current culture |
| 4.6 dog committee | exchange with the committee on application, other exchange with the committee; everything that explicitly relates to the dog committee |
| 5. inductive codes |  |
| 5.1 cleaning staff | relationship with cleaning staff, encounters, relationships, challenges with cleaning staff |
| 5.2 communication | addressing problems with each other, dealing with each other (regardless of dogs) |
| 5.3 characteristics dog |  |
| 5.3.1 «loud» characteristics | excited, loud, enthusiastic, energetic, curious, etc., barking behavior |
| 5.3.2 «calm» characteristics | reserved, quit, anxious, cautious, etc. |
| 5.3.3 dealing with other animals | dealing with other dogs and other animals |
| 5.3.4 dealing with people | dealing with people, also with dog owner |
| 5.3.5 other | coat, origin, handling of objects, indoor/outdoor preferences, eating behavior |
| 5.4 characteristics interviewee | characteristics, personal convictions, general attitude |
| x. important statements |  |
| x.1 mentally away from the workplace | mentally away from the workplace, also when so distracted that interviewee is almost too late |
| x.2 social (teamwork) | teamwork, mutual interaction between work colleagues |
| x.3 dogs’ influence on job satisfaction | role of the dog in the choice of job, influence on job satisfaction |
| x.4 clinic as a specific workplace with dog | benefits of dogs in the workplace, regarding the dog itself, clients, but also concerns about it |
| x.5 disagreements (office vs. outdoor kennel) | disagreements, concerns about dogs’ location |
| x.6 disregard of rules | disregard of rules by dog owners, also regarding communication by the dog committee |
